# Tracking the evolution of a cold stress associated gene family in cold tolerant grasses

**DOI:** 10.1186/1471-2148-8-245

**Published:** 2008-09-05

**Authors:** Simen R Sandve, Heidi Rudi, Torben Asp, Odd Arne Rognli

**Affiliations:** 1 Department of Plant and Environmental Sciences, University of Life Sciences, Ås, Norway; 2 Department of Genetics and Biotechnology, University of Aarhus, Forsøgsvej 1, 4200 Slagelse, Denmark

## Abstract

**Background:**

Grasses are adapted to a wide range of climatic conditions. Species of the subfamily Pooideae, which includes wheat, barley and important forage grasses, have evolved extreme frost tolerance. A class of ice binding proteins that inhibit ice re-crystallisation, specific to the Pooideae subfamily lineage, have been identified in perennial ryegrass and wheat, and these proteins are thought to have evolved from a leucine-rich repeat phytosulfokine receptor kinase (*LRR-PSR*)-like ancestor gene. Even though the ice re-crystallisation inhibition function of these proteins has been studied extensively *in vitro*, little is known about the evolution of these genes on the molecular level.

**Results:**

We identified 15 putative novel ice re-crystallisation inhibition (IRI)-like protein coding genes in perennial ryegrass, barley, and wheat. Using synonymous divergence estimates we reconstructed the evolution of the IRI-like gene family. We also explored the hypothesis that the IRI-domain has evolved through repeated motif expansion and investigated the evolutionary relationship between a LRR-domain containing IRI coding gene in carrot and the Pooideae IRI-like genes. Our analysis showed that the main expansion of the IRI-gene family happened ~36 million years ago (Mya). In addition to IRI-like paralogs, wheat contained several sequences that likely were products of polyploidisation events (homoeologs). Through sequence analysis we identified two short motifs in the rice *LRR-PSR *gene highly similar to the repeat motifs of the IRI-domain in cold tolerant grasses. Finally we show that the LRR-domain of carrot and grass IRI proteins both share homology to an *Arabidopsis thaliana *LRR-trans membrane protein kinase (*LRR-TPK*).

**Conclusion:**

The diverse IRI-like genes identified in this study tell a tale of a complex evolutionary history including birth of an ice binding domain, a burst of gene duplication events after cold tolerant grasses radiated from rice, protein domain structure differentiation between paralogs, and sub- and/or neofunctionalisation of IRI-like proteins. From our sequence analysis we provide evidence for IRI-domain evolution probably occurring through increased copy number of a repeated motif. Finally, we discuss the possibility of parallel evolution of LRR domain containing IRI proteins in carrot and grasses through two completely different molecular adaptations.

## Background

The Poaceae family (grasses) contains some of the most economically important and well studied plant species, e.g. maize, wheat, barley, and rice. Generally speaking the Pooideae subfamily, which includes wheat, barley and forage grasses, are adapted to cold seasons. Many species in this subfamily can withstand temperatures far below freezing and intercellular ice formation [1,2]. Rice and maize on the other hand belongs to the subfamilies Ehrhartoideae and Panicoideae, respectively, and are adapted to warm and tropical climates. Pooideae lineage (from now on referred to as cold tolerant grasses) adaptation to cold climates makes grasses an interesting model system for studying climatic adaptation at the physiological and molecular level.

Frost tolerance adaptations are in many organisms associated with the evolution of antifreeze proteins (AFPs) [3]. AFPs can affect freezing- and ice crystallisation related stress via different mechanisms. Thermal hysteresis (TH) depresses the freezing point at which ice crystallisation initiates, which render it possible for organisms to survive under freezing temperatures. Ice re-crystallisation inhibition (IRI) on the other hand does not hinder ice crystallisation but manipulates the growth of the ice crystals such that small ice crystals grow at the expense of larger ice crystals, and this has been suggested to prevent or minimize the cellular damage in plants [4]. A third mode of AFP action is membrane stabilisation which has been reported for a fish AFP [5]. Animal AFPs generally possess high thermal hysteresis (TH) characteristics and lower ice crystallisation initiation temperature by 1–5°C [6,7]. Plant AFPs on the other hand have low TH-activity, but exhibits strong ice re-crystallisation inhibition (IRI) activity [6].

Genes encoding peptides with IRI capacity have evolved independently several times in different lineages of higher plants. These IRI peptides are homologous to diverse protein classes, e.g. thaumatin like proteins, endochitinases, endo-B-1,3-glucanase, and leucine rich repeat (LRR) containing proteins [6,8,9]. Three LRR-domain containing IRI proteins (LRR-IRI) have been identified in plants, one in carrot (DcAFP; accession number AAC6293) and two in wheat (TaIRI1 and TaIRI2 with accession numbers AAX81542 and AAX81543) [10,11]. DcAFP has been classified as a polygalacturonase-inhibiting protein (PGIP) but does not exhibit PGIP activity [12]. LRR motifs span across the entire processed DcAFP protein and form 10-loop beta-helix secondary structure with solvent exposed asparagine residues at putative ice binding sites [13]. *TaIRI1 *and *TaIRI2 *genes (accession numbers AY9968588 and AY968589) have been identified as homologous to the LRR-domain coding region of a rice phytosulfokine LRR receptor kinase (*OsLRR-PSR*: NP_001058711) and an *Arabidopsis *trans-membrane protein kinase (*AtLRR-TPK*: NP_200200). The wheat IRI peptides differ structurally from DcAFP in that the LRR-domain only comprises about half of the processed peptide [10].

In addition to the N-terminal LLR domain, wheat IRI proteins have a C-terminal repeat domain consisting of two similar A and B motifs, NxVxG and NxVxxG, respectively. This repeat domain has been reported to exhibit strong *in vitro *IRI capacity [14]. Interestingly, blast search yields no sequences with homology to the IRI-domain outside the subfamily of cold tolerant grasses [10]. Protein modelling has shown that the A and B repeated motifs of the IRI-domain folds into a B-roll with ice binding sites matching the prism face of ice [15]. Expression studies have shown that increased expression levels in wheat [10] and perennial ryegrass [Rudi et al, unpublished] are correlated to cold acclimation, but no *in vivo *studies to determine the localisation of these grass IRI peptides have been reported in the literature. However, *TaIRI1 *and *TaIRI2 *have been predicted to encode a N-terminal 20 amino acid signal peptide domain targeting the proteins to the secretory pathway, suggesting that the peptides could be located in the extracellular space [10].

While *DcAFP *is evolutionarily closely related to PGIPs; *TaIRI1 *and *TaIRI2 *genes are thought to have evolved from a LRR-PSR like ancestor gene. Furthermore, the evolutionary origin of the IRI-domain in grass IRI genes is much less obvious, because the IRI-domain is not homologous to any other sequences outside the cold tolerant grass lineage. Tremblay et al. [10] proposed a "TE-hypothesis" to explain this apparent lack of homologous coding regions; that the IRI-domain had arisen by a transposable element (TE) insertion. However, no TE signature sequence could be identified surrounding the IRI-domain [10], thus no empirical data supports the TE-hypothesis so far.

Here we report the identification and characterisation of novel LRR-IRI homologous genes in cold tolerant grass species. We perform a detailed study of the evolutionary relationships between *OsLRR-PSR *and IRI-like genes by analysing sequence divergence at synonymous sites. We also use synonymous site divergence to trace the evolutionary history of the IRI-like gene family with respect to gene duplication events. The evolution of gene families *per se *is in itself a much debated topic, and gene family expansion and subsequent functional diversification is thought to have been a significant factor contributing to adaptations to new environments [16,17]. The evolution of the IRI-like gene family of cold tolerant grasses is discussed in the context of the Duplication-Degeneration-Complementation (DDC) model [18]. Finally we address the unresolved matter of the evolutionary mechanism underlying the birth of the cold tolerant grass IRI-domain, and propose a novel hypothesis on the evolution of this IRI-domain.

## Results

### Screening of perennial ryegrass BAC libraries

Initial screening of the perennial ryegrass (*Lolium perenne*) LTS18 BAC library with the *LpAFP *primer pair produced two hits, from which *LpIRI1 *(EU680848) and *LpIRI2 *(EU680849) were isolated. The NV#20F1-30 BAC library produced four hits with the LpIRIx primer pair, and three hits with the LpAFP primer pair. *LpIRI4 *(EU680851) was subsequently isolated from one of the four positive LpIRIx hits and *LpIRI3 *(EU680850) was isolated from one of three positive LpAFP hits. All genes isolated from perennial ryegrass were intronless and encoded putative peptides with high identity to the wheat *TaIRI1 *and *TaIRI2 *genes (blastp < 4e-10). *LpIRI1*, *LpIRI4*, and *LpIRI3 *were similar in size and encoded peptides of 285, 242, and 254 amino acids, respectively. *LpIRI2 *encoded a shorter ORF of 150 amino acids that was 94% identical to the LpIRI4 IRI-domain. The IRI-domain of LpIRI3 is identical to an earlier identified partial IRI peptide encoded by *LpAFP *(AJ277399).

Nucleotide alignments of the LpIRI-like genes showed that *LpIRI2 *has undergone a deletion of almost the entire LRR-domain coding region, the only remains of it being a 102 base pair (bp) fragment upstream of the *LpIRI2 *putative start codon. This could indicate that *LpIRI2 *is a pseudogene or a non-functional allele. Non-functional sequences are expected to evolve under neutral expectation, which means that the rate of non-synonymous to synonymous substitutions (w) is expected to be 1. Average w between *LpIRI2 *and the other perennial ryegrass sequences was estimated to be 0.56 which suggests that *LpIRI2 *is under selective constraints despite the major deletion in the LRR-domain.

### *In silico *identification of IRI-like sequences

The blastn EST search resulted in 189 wheat, 100 barley, 21 tall fescue (*F. arundinacea*), 5 Italian ryegrass (*L. multiflorum*) and 2 darnel ryegrass (*L. temulentum*) sequences. *In silico *mining produced from zero to eleven full length IRI-like sequences (i.e. with a start and stop codon) per species (Table 1), and sequences were annotated as follows; an initial two letters indicating latin species name, "C" indicating a contig of more than two ESTs, and lastly an identifier number. The number of ESTs in the contigs ranged from 2 to 40 (Table 2), with an average of 17 ESTs per contig. In addition we identified several partial IRI-like sequences; one partial wheat IRI-like contig of 10 ESTs, and three partial barley sequences of 2, 3, and 6 EST sequences. The partial sequences were not included in further analysis. A barley mRNA, AK249041, did not align to any barley contig but coded for a full length IRI-like ORF, hence we included this mRNA in our dataset. *In silico *mining with tall fescue, Italian ryegrass, and darnel ryegrass ESTs did not produce any full length IRI-like sequences. *In silico *EST mined sequences were considered validated if a core nucleotide sequence from NCBI had > 99% identity over > 200 bp. TaC3 was validated as being identical to *TaIRI1*, and HvC1 was validated by being identical to a full length mRNA sequence (AK252915). Accession numbers for ESTs belonging to full length IRI-like contigs acquired through *in silico *mining are included in additional material [see Additional file 1].

**Table 1:** All IRI-like sequences identified through EST *in silico *mining.

Species	No. EST sequences	IRI-like sequences	
		
		Full length	Partial
Wheat	189	11	1
Barley	100	3	3
Tall fescue	21	0	2
Italian ryegrass	5	0	1
Darnel ryegrass	2	0	1

IRI-like gene homologous ESTs were identified by a blastn search using *TaIRI1*.

**Table 2:** Full length IRI-like sequences identified through EST *in silico *mining and the number of ESTs per contig.

Species	Contig name	ESTs in contig
Wheat	TaC1	16
	TaC2	39
	TaC3	19
	TaC4	21
	TaC5	16
	TaC6	12
	TaC7	16
	TaC8	17
	TaC9	3
	TaC10	4
	TaC11	2
Barley	HvC1	23
	HvC2	40
	HvC3	15

### Predicted protein structure characterisation

**Figure 1:**
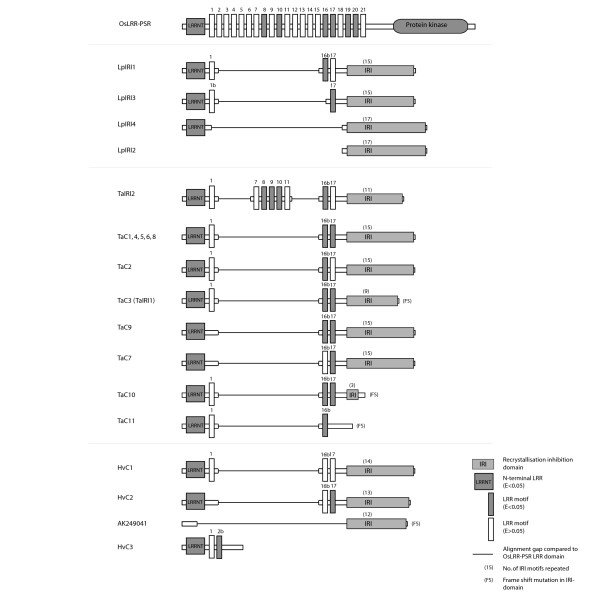
**Predicted peptide structure of *OsLRR-PSR *and IRI-like sequences**. Peptide structures of the LRR and IRI-domains of IRI-like sequences as predicted by Pfam and by visual characterisation, respectively. *OsLRR-PSR *was used as a template for peptide structure comparisons. Grey LRR-domains were predicted with significant P-values in Pfam (P < 0.05). Numbers of IRI motifs in the IRI-domains and whether frame shift mutations were identified are given in parentheses.

The *OsLRR-PSR *peptide sequence was used as a template for comparisons of the LRR-domains. The *OsLRR-PSR *is suitable for this purpose because it is a putative homologue to the LRR-domain of the IRI-like sequences. In the following peptide structure characterization we assume that the most recent common ancestor (MRCA) of all IRI-like grass genes encoded the same domain architecture as *OsLRR-PSR*. *OsLRR-PSR *was predicted to encode one LRR N-terminal domain (LRR-NT), 21 internal LRR homologous motifs (1–21), and a protein kinase (PK) C-terminal domain. Only the LRR-NT and six internal LRR motifs were predicted with significant E-values by Pfam. Compared to *OsLRR-PSR *all predicted IRI-like peptides have reduced number of LRR motifs and lacks the PK-domain. Three internal LRR motifs did not align to any OsLRR-PSR LRR motifs. These have probably arisen through deletions in the LRR-domain causing two partial LRR motifs to merge into a novel LRR motif. Comparative sequence analysis suggests that deletions between LRR motifs 1–16, 2–16, and 12–16 of a *OsLRR-PSR*-like grass ancestor gene have resulted in three novel LRR-domains; LRR1b, 2b, and 16b (Fig. 1).

The IRI-domain also varies in size by number of repeated motifs (Fig. 1). About 60% of all sequences with an IRI-domain have 15 repeat motifs or more. Six sequences were detected to have a reduced number of repeat motifs, or had completely lost the IRI-domain. Analysis of codon based nucleotide alignments revealed that frameshift (FS) mutations could be identified in four of the IRI-like sequences (TaC3, TaC10, TaC11, and AK249041) that showed reduced IRI-domain size (data not shown). HvC3 is the only IRI-like sequence with a completely reduced IRI-domain. For all sequences in the HvC3 contig additional information on abiotic conditions under which the plants had been grown were included in the EST files. Without exception all ESTs originated from tissue sampled from etiolated barley seedlings, and not from cold acclimated tissue. This is congruent with HvC3 lacking the entire ice binding IRI-domain, suggesting that IRI-like paralogs are involved in several different stress responses.

Prediction of the subcellular location of the IRI-like peptides (see methods) predicted a signal peptide that targets the peptides to the secretory pathway present in all IRI-like peptides, except from LpIRI2. The lack of an LpIRI2 signal peptide, and the fact that LpIRI2 has undergone a deletion of almost the entire LRR-domain could suggest that *LpIRI2 *is in fact a non-functional allele or pseudogene. However the results from the w estimates contradict the non-functionality hypothesis. Alternatively the lack of a signal peptide can be interpreted as that LpIRI2 simply has evolved a different function than the IRI-like peptides with a conserved signal peptide.

### Phylogenetic analysis of IRI-like paralogs

**Figure 2:**
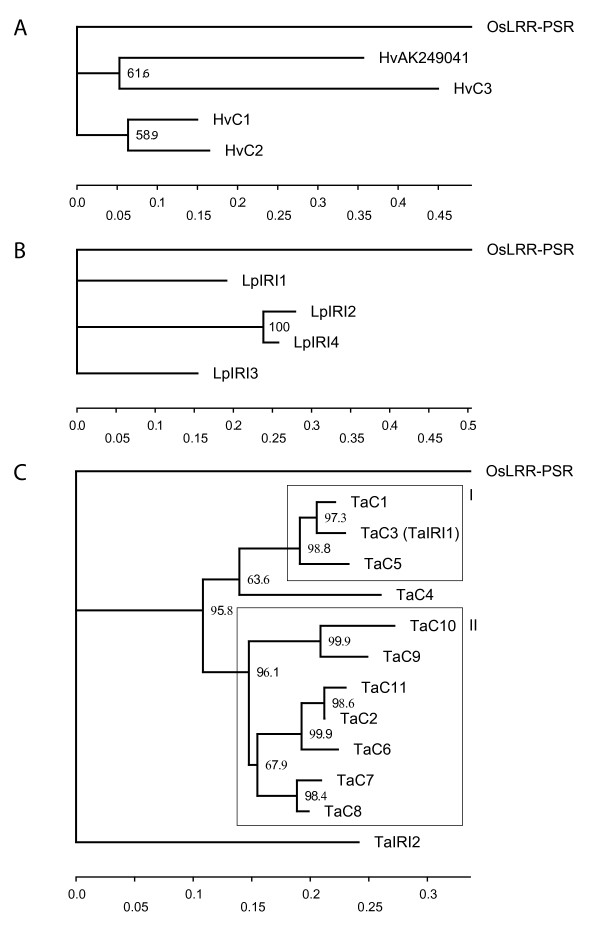
**Maximum Likelihood paralog phylogenies of all full length IRI-like amino acid sequences**. Bootstrap values from 1000 replicates are in percent at internal nodes. Bootstrap values < 50 are not shown. A) Phylogeny of barley IRI-like full length sequences. B) Phylogeny of perennial ryegrass IRI-like full length sequences. C) Phylogeny of wheat IRI-like full length sequences. Two monophyletic clades of very low within-clade synonymous divergence (I, II) are boxed.

*OsLRR-PSR *has highest homology to IRI-like genes outside the Pooideae subfamily (blastp E-value of 2e^-46^) and was therefore used as an out-group in the phylogenetic analysis. The paralog phylogenies of perennial ryegrass, barley, and wheat IRI-like peptide sequences are given in Figure 2A, 2B, and 2C, respectively. In the wheat phylogeny TaIRI2, the amino acid sequence with the highest number of conserved LRR-domains, diverge as a monophyletic branch, while all other wheat sequences with further reduced LRR-domain sizes form a second monophyletic group. This large monophyletic group can be subdivided into two smaller monophyletic clades (Fig. 2C). In group I a FS mutation in the IRI-domain of TaC3 shortens the ORF and separates TaC3 structurally from the other sequences. One might be tempted to speculate if this FS is due to alignment errors but the FS in the TaC3 contig have been validated independently by the full length mRNA *TaIRI1*. Group II contain IRI-like genes with high degree of divergent predicted peptide structure, i.e. putative peptides with strongly reduced and completely lost IRI-domain (TaC9 and TaC11) as well as predicted peptides with large IRI-domains. Alignments of predicted amino acid sequences with all FS mutations removed, that were used for the phylogenetic analysis, are presented in additional material [see Additional file 2, 3, and 4].

### Estimation of synonymous divergence of IRI-like sequences

If we assume a molecular clock, synonymous substitution rates (dS) between two DNA sequences can be interpreted as a relative measurement of time since MRCA, thus for two paralogous genes dS can be interpreted as the time since gene duplication [19]. Without being able to account for all the IRI-like paralogs existing in a genome we cannot infer if two paralogs descend from a single duplication event (i.e. being true paralogs) or if they are products of two separate duplication events. We therefore restricted our initial analysis of IRI-like gene duplication events to only comprise the dS_max _and dS_min _for all pairwise comparisons. The dS_max_-dS_min _range can be interpreted as the evolutionary time span in which all duplications of IRI-like genes in our dataset have occurred.

**Figure 3:**
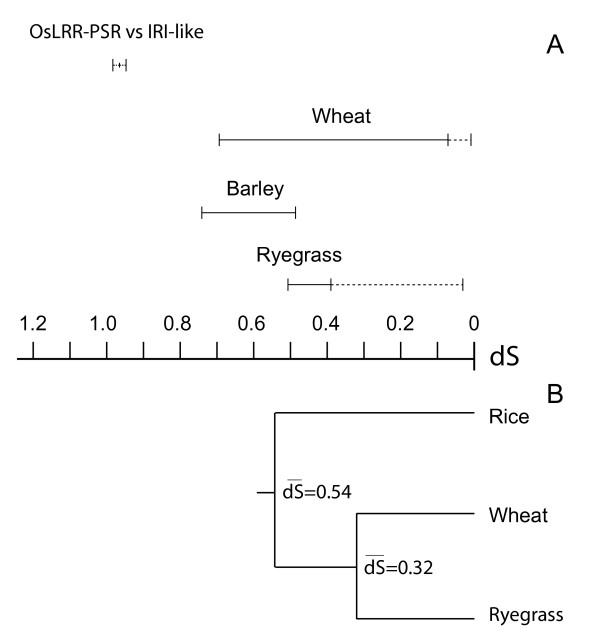
**Synonymous divergence between paralogous and orthologous sequences**. A) Maximum and minimum dS range between all IRI-like paralogous sequences of wheat, barley and perennial ryegrass and the average dS between *OsLRR-PSR *and all IRI-like sequences. Dotted lines for the divergence estimate between OsLRR-PSR and IRI-like sequences represent SD of the ortholog dS. Dotted lines for wheat and perennial ryegrass indicate changes in dS range if putative alleles are included. B) A tree indicating the average synonymous divergence, given in dS, estimated from ten control genes from rice, wheat, and perennial ryegrass.

The average dS between *OsLRR-PSR *and LRR-domains of all IRI-like sequences from cold tolerant grasses was estimated to 0.97 (standard deviation (SD) = 0.11). Hence if *OsLRR-PSR *is the true ortholog of IRI-like genes, the radiation of cold tolerant grasses from rice predates the initiation of IRI-like gene family expansion in our dataset (Fig. 3A). All paralog dS ranges for wheat, barley and perennial ryegrass overlapped, however there are large range differences. These range differences is caused by the low dS_min _range of wheat and perennial ryegrass IRI-like sequences (Fig. 3A). For perennial ryegrass the dS between *LpIRI2 *and *LpIRI4 *lowered the dS_min _estimate from 0.40 to 0.03. Dissection of wheat paralog dS estimates showed that the low wheat dS_min _range could be traced back to low pairwise dS within the monophyletic clades I and II (Fig. 2C). If dS estimates from sequence pairs within clade I and II were removed the wheat dS_min _shifted from 0.01 to 0.39.

Very low dS between paralog pairs might reflect the inclusion of highly diverged alleles in our paralog dataset. Sequencing errors in ESTs and inclusion of highly diverged genotypes in our dataset could potentially give an inflated polymorphism level producing artificial contigs that are alleles rather than paralogs. To identify putative false paralogs we set an allelic dS threshold of dS < 0.03 (see methods section). Based on this definition we identified two putative allelic sequence pairs TaC2-TaC11 (dS = 0.01) and *LpIRI2*-*LpIRI4 *(dS = 0.03).

### Estimation of synonymous divergence between control genes

The ten control orthologs chosen for evolutionary rate control and their pairwise dS estimates are listed in Table 3. Based on these genes the average dS between rice and cold tolerant grasses were estimated to be 0.54 with an average SD between dS estimation methods of 0.08. The estimated divergence of rice and cold tolerant grasses based on the control genes (dS = 0.54) is substantially lower than the minimum equivalent estimate found by using *OsLRR-PSR *and IRI-like genes (dS = 0.72) (Fig. 3A and 3B). The average dS between perennial ryegrass and wheat genes were 0.32, with an average SD of 0.04 between the estimation methods.

**Table 3:** Evolutionary rate control genes and their pairwise synonymous distances.

Gene	Accession number			Synonymous distance (dS)	
	
				Ta vs Lp	TaLp vs Os
	Ta	Lp	Os	dS	dS*
Cytosolic glyceraldehyde-3-phosphate dehydrogenase	EF592180	EF463063	NM_001059674	0.33 (0.04)	0.47 (0.06)
Actin	AB181991	AY014279	NM_001062196	0.30 (0.03)	0.49 (0.07)
Gibberellin 20-oxidase	Y14008	AY014281	NM_001058486	0.37 (0.02)	0.70 (0.13)
Phytochrome B	AF137331	AF137308	NM_001056445	0.39 (0.04)	0.57 (0.07)
Casein protein kinase 2 alpha subunit	AB052133	AB213317	NM_001065287	0.30 (0.05)	0.48 (0.09)
Na+/H+ antiporter precursor	AY461512	AY987047	NM_001074903	0.33 (0.04)	0.58 (0.09)
Putative plasma membrane Na+/H+ antiporter	AY326952	AY987046	CB634542	0.14 (0.02)	0.44 (0.06)
Myo-inositol phosphate synthase	AF542968	AY154382	NM_001055777	0.32 (0.04)	0.57 (0.07)
Cinnamoyl CoA reductase	CK161291	AF010290	NM_001052667	0.28 (0.01)	0.41 (0.03)
Fructan beta-(2,1) fructosidase	AJ564996	DQ016297	NM_001052039	0.39 (0.06)	0.72 (0.11)

Accession numbers are given for putative orthologous genes from wheat (Ta), perennial ryegrass (Lp) and rice (Os) used for evolutionary rate control. Their synonymous pairwise divergence was calculated as a mean of three estimates (see methods section), and the standard deviation of all three estimates is given in parenthesis. *denotes a mean synonymous distance of two pairwise comparisons between perennial ryegrass-rice and wheat-rice (referred to as TaLp-Os).

### Molecular analysis of LRR and IRI-domains

**Figure 4:**
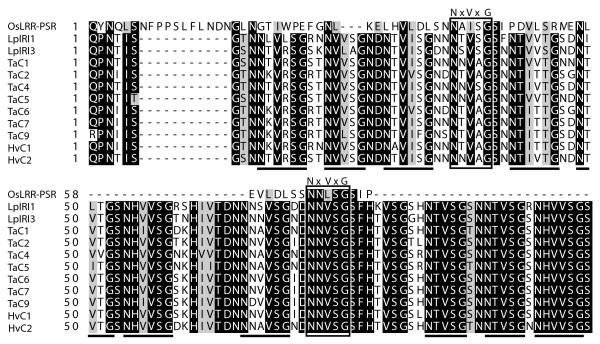
**Amino acid alignment between IRI-like sequences and *OsLRR-PSR *at the initiation of the IRI-domain**. Shaded residues are identical in > 90% of sequences. Black bars underlines IRI-domain A- and B motifs (NxVxG/NxVxxG). Boxed motifs are IRI-domain motifs shared between OsLRR-PSR and IRI-like sequences.

The alignment of the LRR-domain of *OsLRR-PSR *and the IRI-domains of a subset of the IRI-like peptide sequences shows two blocks of strikingly high conservation between the OsLRR-PSR and two IRI-domain motifs (Fig. 4). The two motifs have 2/3 of the motif sequence residues conserved between rice and cold tolerant grasses. The first motif has an Ile-Val substitution while the second motif has a Leu-Val substitution, neither of which are substitutions with large effect on hydrophilic properties.

**Figure 5:**
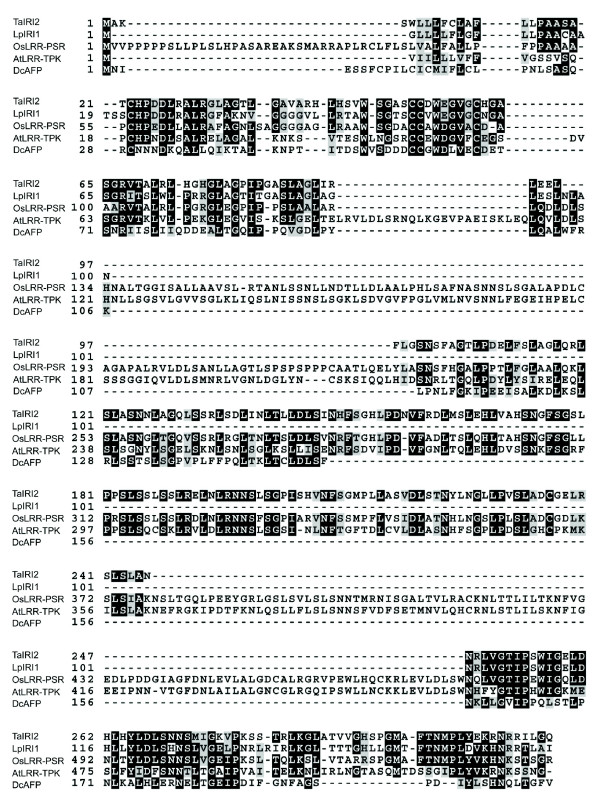
**Amino acid alignment of homologous LRR-domains from monocot and dicot species**. The alignment shows homologous LRR-domains between Arabidopsis (AtLRR-TPK), carrot (DcAFP), rice (OsLRR-PSR), wheat (TaIRI1) and perennial ryegrass (LpIRI1). All sequences in the alignment has a blastp E-value of > 1*10^-18 ^to at least one other sequence in the alignment. Shaded residues are identical in > 60% of sequences.

Relatively low level of conservation between DcAFP and grass IRI-like peptide sequences was found (blastp 1e-4). All sequences in the LRR-domain alignment (Fig. 5) have a blast E-value of < 1e-18 to at least one other sequence in the alignment, but no larger blocks of conservation between DcAFP and any monocot LRR-domain can be identified. However we observe that several blocks of 2–4 conserved residues exist throughout the alignment of the LRR-domain between DcAFP and grass IRI-sequences.

## Discussion

Until now only three cold tolerant grass IRI protein coding genes have been reported; a partial coding sequence of an IRI-domain from perennial ryegrass [14], and two highly identical full length mRNA paralogs from wheat [10]. Through *in silico *mining and BAC sequencing we have identified 15 full length and 8 partial novel IRI-like genes in cold tolerant grasses. In addition, we have obtained the complete sequence of *LpAFP*. The data accumulated leaves no doubt: cold tolerant grasses of the Pooideae subfamily have evolved a lineage specific family of IRI-like genes.

### IRI-gene family radiation happened after the cold tolerant grass divergence

The prevailing hypothesis on the evolution of LRR-IRI-like genes belonging to cold tolerant grasses is that they are lineage specific and that an *OsLRR-PSR*-like gene is the MRCA [10]. This hypothesis was proposed based on sequence homology data only and we therefore re-examined this idea using more rigorous statistical methods by estimating synonymous divergence. When employing a commonly used mutation rate for grasses of 6.5*10^-9 ^[20-22], estimated by Gaut et al. [23], the synonymous divergence level between *OsLRR-PSR *and IRI-like sequences suggested a MRCA about 75 Mya. This is slightly higher than upper thresholds of some published rice-Pooideae divergence estimates [22]. However, our estimate of divergence time between rice and cold tolerant grasses based on the control genes suggest a rice-Pooideae divergence only 42 Mya. This is similar to divergence estimates published by Patterson et al. [24] and Salse et al. [20], dating back 41–47 and ~46 Mya, respectively. Our wheat and perennial ryegrass divergence estimates is dated ~10 million years prior to a previously published estimate of ~35 Mya [25].

The observed discrepancy between the two divergence estimates of rice and cold tolerant grasses in our study (Fig. 3A and 3B) can be interpreted in two different ways. The *OsLRR-PSR *gene is the true ortholog of IRI-like genes and the incongruent divergence time estimates are caused by differences in molecular clock rates. Or alternatively, if the molecular clock rate is similar, it follows that *OsLRR-PSR *diverged from IRI-like genes long before rice and cold tolerant grasses diverged (Fig. 3). The burst of IRI-like sequence duplications must then have occurred in the ancestor genome of rice, and this implies that the rice genome subsequently must have lost all genes belonging to the IRI-like gene family. Even though loss of genes and whole gene families is not an uncommon feature of plant genome evolution [16,24], elevated clock rate differences is a more parsimonious explanation to the divergence estimate differences seen in Figure 3A and 3B. Evolutionary rate differences are highly common among closely related species, different lineages of a species, and also within a genome [26,27]. When using a single gene family to estimate divergence between species, as with the IRI-like genes, deviation from the average genome clock rate would be expected. As an example the clock rate of the ten control genes varied from 4.1–7.1*10^-9^, with an average rate of 5.4*10^-9^, when using a divergence time between rice-cold tolerant grasses of 50 My.

Assuming true orthologous relationship between *OsLRR-PSR *and IRI-like genes we can calibrate an average molecular clock rate for IRI-like genes using the dS = 0.97 and assuming an absolute divergence time of 50 Mya (see methods). This gives us an estimate of an IRI-like gene family specific clock rate of 9.7*10^-9^. Employing this adjusted clock rate pushes the estimates for the initiation of IRI-like gene duplications forward to 36, 27, and 39 Mya for wheat, perennial ryegrass and barley, respectively. This is approximately 3–14 My after our estimate for divergence between rice and cold tolerant grasses based on the control genes.

### Species specific differences in IRI-like sequence numbers

Twice as many IRI-like sequences, partial and full length, were identified in wheat compared to barley (Table 1). *In silico *mining is vulnerable to methodologically introduced uncertainties. For example, the fact that the wheat EST database at NCBI is more than twice as large (1.2 M ESTs) than for barley (500 K ESTs) could be a contributing factor to the differences in numbers of IRI-like mined sequences because we expect that the EST database size is positively correlated to transcriptome coverage of an organism. A separate effect of a larger EST database will be the inclusion of ESTs from an increased number of genotypes, which could be a source of introduction of allelic polymorphisms.

Even though methodological properties might elevate the number of wheat sequences identified to some extent, we believe that much of the difference in wheat and barley IRI-like sequence numbers are related to genomic ploidy level differences. Wheat (*Triticum aestivum*) is an allo-hexaploid originating about 8.000 years ago. It has three homoeologous genomes A, B, and D, which are estimated to have diverged 4.5–2.5 Mya [25]. Our results from the phylogenetic analyses, supported by the dS estimates, suggests that IRI-like sequences within monophyletic clade I and II (Fig. 2) could be homoeologous rather than paralogous. But low pairwise dS can alternatively reflect recent gene duplications. Consequently our inferences on the evolutionary relationship between putative wheat homoeologous sequences must be viewed in a critical manner.

### A model of wheat IRI-like sequence evolution

**Figure 6:**
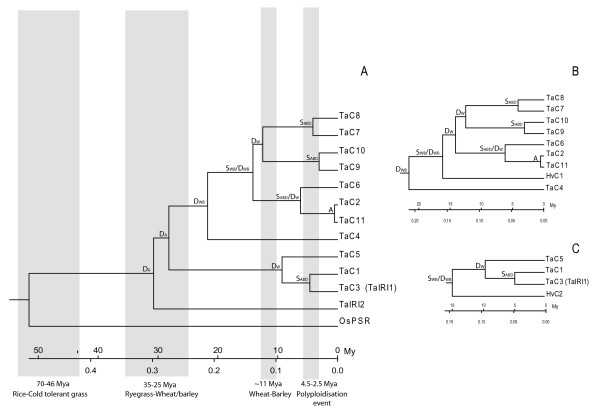
**Evolutionary relationships between the wheat IRI-like sequences inferred with an IRI-like gene specific molecular clock rate**. Phylogenetic trees based on pairwise synonymous distances made with Unweighted Pair Group Method with Arithmetic Mean (UPGMA). Distance scale shows synonymous site divergence and estimated absolute divergence time in million years. Internal nodes represent duplication, speciation, or allelic divergence events. D_A _= ancient duplication events shared by wheat, barley, and perennial ryegrass; D_WB _= duplication events shared by wheat and barley; D_W _= duplication events exclusive for the wheat lineage; S_WB _= speciation event between wheat and barley; S_ABD _= speciation event for the A, B, and D genomes of wheat; A = allelic divergence event. A) Tree based on all wheat IRI-like sequences. B) Sub-tree of monophyletic clade II including the two most recently radiated IRI-like sequences in our dataset (HvC1 and TaC4). C) Sub-tree of monophyletic clade I including the single most recently radiated barley IRI-like sequence.

As mentioned, wheat differs from the diploid grasses in our study in that it has many more and younger IRI-like paralogs, some of which probably can be accounted for by wheat polyploidisation. Using the IRI gene specific clock rate of 9.7*10^-9 ^we built a model of the evolutionary history of wheat IRI-sequences using a phylogenetic approach (Fig. 6A). According to literature and the data acquired in this study wheat and barley specific duplications would be younger than ~35-25 My and wheat specific duplications would be younger than ~11 My [25]. The internal nodes of the phylogeny in Fig. 6 can therefore be interpreted as putative ancient duplication events happening before divergence within Pooideae (D_A_), putative duplication events specific to wheat and barley (D_WB_), putative speciation event between wheat and barley (S_WB_), putative gene duplication events specific to wheat (D_W_), speciation events of the A, B, and D genomes of wheat (S_ABD_), and lastly allelic divergence (A).

The evolutionary model with adjusted clock rates strengthens our hypothesis on the homoeologous relationship of the wheat clade I and II sequences (Fig. 2). The divergence between TaC6 and TaC2/TaC11 are predicted slightly earlier than the polyploidisation event, thus this internal node could not be classified unambiguously. Sub-trees of clade I and II, in which we have included the barley sequence with lowest synonymous distance to each clade, are presented in Figure 6B and 6C, respectively. The sub-trees further support the hypothesis that clade I and II represents genes that mainly have arisen through polyploidisation events. In both sub-trees the closest related barley sequence is estimated to have diverged from the wheat clade I and II about 15 Mya, coinciding with wheat-barley divergence [25]. However without a complete knowledge of the orthologous relationships of the IRI-like sequences the inferences on evolutionary relationships are somewhat speculative.

### Structural and functional diversification of the IRI-like gene family

A striking feature of the IRI-like gene family is the structural differentiation between paralogs (Fig. 1). Structural diversification of IRI-like genes, as seen in our sequence collection, would be expected to affect the spectrum of IRI-like peptide function, because both LRR and IRI-domains are known to be involved in substrate binding [15,28]. One interpretation of this pattern is that IRI-like sequences with complementary combinations of LRR motifs and IRI-domain sizes are selected for and retained in the genome, which is what we expect from the duplication-degeneration-complementation (DDC) model of paralog evolution [18]. DDC predicts that mutations in regulatory elements increase the probability of paralog retention because it leads to partitioning of ancestral functions (subfunctionalisation), and the model has proven to be an important contribution to understanding evolution of paralogous genes [29,30]. The DDC model has later been expanded to coding sequences [31,32], and recently a combination of regulatory and structural DDC has been demonstrated [33,34].

Regulatory subfunctionalisation in gene expression and tissue localisation has been demonstrated between *TaIRI1 *and *TaIRI2 *(TaC3) [10], two genes coding for highly divergent LRR-domain structure and length. In our study we have also found evidence that peptide structure divergence has led to sub- or neofunctionalisation. A barley IRI-like sequence contig (HvC3) with no IRI-domain still seems to play a functional role under etiolation. This suggests that the LRR-domain of IRI-like genes may play a functional role in multiple stress responses. Other LRR-domain containing genes in plants have also been shown to be involved in stress responses under drought stress and as a key membrane-bound regulator of absiscic acid signalling [35,36].

One interesting aspect of the structural divergence pattern is that all genes except *LpIRI2 *are predicted to encode a conserved N-terminus signalling domain targeting the proteins to the secretory pathway. Secretion to the apoplast is expected for proteins with ice interacting functions. In the light of these data, an interpretation of the structural variability of LRR-domains, combined with the apparent conservation of the N-terminal signalling domain, is that IRI-like genes might be under selective pressure for a continuous ORF from the signalling domain across the LRR-domain and into the IRI-domain, conserving the crucial function of apoplast export of IRI peptides. The LRR-domain itself might not be under functional conservation. As an example: the full length sequenced mRNA AK249041 from barley has a N-terminal conserved predicted signal peptide motif, a completely reduced LRR-domain with no predicted LRR motifs, and an IRI-domain.

Less dramatic polymorphisms between paralogs, such as single amino acid substitutions or small motif number differences could potentially have a large effect on the functionality. Single amino acid substitutions have been shown to radically change AFP functionality in both plant and animal AFPs [13,37]. Chakrabartty and co-workers [38] showed that only small deletions in an AFP with repetitive structure from flounder altered the ice interacting properties dramatically. Thus, all the observed polymorphisms between IRI paralogs, even down to single amino acid substitutions, could potentially be of functional significance.

### Birth of an IRI repeat domain

The molecular mechanisms underlying the metamorphosis from an *OsLRR-PSR*-like ancestor gene into the first bipartite IRI-like gene have been addressed by Trembley et al. [10]. They proposed the "transposable element hypothesis" (TE) suggesting that the IRI-domain is a TE insertion that has resulted in a FS mutation and caused the loss of the PK-domain. However no TE signatures were found flanking the IRI-domain [10]. Based on results from our sequence analysis (Fig. 4) we propose a competing hypothesis on the evolution of the IRI-domain, namely the repeated motif expansion (RME) hypothesis. It has been shown that expansions of domains by duplication of repeated motifs are common in genes of repetitive structure [39]. We suggest that IRI motifs have increased in copy number by a yet unknown mechanism, possibly illegitimate recombination, slippage, or uneven crossing over. Contrary to the TE-hypothesis the RME hypothesis can explain the evolution of the IRI-domain and at the same time account for the existence of two IRI motif-like blocks in *OsLRR-PSR *(Fig 4). Lastly, if the entire IRI-domain is a TE-insertion we would expect this TE sequence to be found at other loci in grasses. However no such reports of TE-like sequences homologous to the IRI-domain are known to our knowledge.

### Convergent evolution of LRR containing AFPs

LRR-domain containing proteins are extremely abundant in plants. The largest LRR containing plant peptide group is LRR receptor kinases (LRR-RK), having more than 200 members in the *A. thaliana *genome [40]. Plant disease resistance associated genes (NBS-LRR) comprise another large LRR containing functional group [41]. Common for the function of LRR domains in any peptide is that they are associated with peptide-peptide recognition and binding interactions [42-44].

Through comparative protein domain analysis we have shown that LRR-domains of IRI-like genes are much less conserved compared to the predicted signal peptide motif flanking the N-terminus of the LRR-domains. We believe that this could be due to lack of selective constrains on the LRR-domain function itself, or perhaps selection for divergent LRR-domain functions as predicted by DDC. Whatever functional role today's IRI-like sequence LRR-domains might play; there is little doubt that the LRR-domain of IRI-like genes in cold tolerant grasses shares an ancient common ancestor with the LRR-domain of DcAFP (Fig. 5). However, while cold tolerant grass IRI-like proteins have evolved ice binding capacity through the evolution of an IRI-domain [14,15], DcAFP have evolved ice binding capacity through changes in the LRR-domain itself [13]. *DcAFP *and grass LRR-IRI genes are therefore intriguing examples of parallel evolution of function by two completely different molecular mechanisms; evolutionary alterations of a pre-existing LRR-domain and evolution of a novel repeat domain with ice binding properties.

## Conclusion

The IRI-like genes identified by Sidebottom et al. [14] and Tremblay et al. [10], and in this study tell a tale of a complex evolutionary history that includes birth of an ice binding domain, a burst of gene duplication events after cold tolerant grasses radiated from rice, domain structure differentiation between paralogs, and sub- and/or neofunctionalisation of IRI-like proteins. Given more detailed functional studies, the IRI-like gene family can provide a valuable example of how duplicated genes evolve novel functional spectres. The hypothesis that evolution of IRI-like genes has been important for Pooideae grass adaptation to cold climate [10] is strengthened by this study as we show that the evolution of the IRI-like gene family probably happened after the divergence from rice, and furthermore that the numbers of IRI-like genes are higher than earlier known.

## Methods

### *In silico *IRI-like sequence mining

A blastn search in the NCBI database was performed using *TaIRI1*. All sequences with blast E-value < 1*10^-20 ^were downloaded from the EST and core nucleotide databases. Contigs were aligned with alignment parameters set to > 97% identity and > 40 nucleotides overlap using Sequencher (Gene Codes Corp., Ann Arbor, MI, USA). The 97% identity threshold was set to allow contig alignments to include different allelic forms and polymorphisms caused by EST sequencing errors. Non-coding nucleotides (i.e. promoter and 3'UTR) were removed after an initial prediction of open reading frame (ORF), and subsequently the sequences were realigned with identical parameters. All contigs were translated into their predicted amino acid sequence. Sequence contigs with lack of start and stop codon due to incomplete sequence coverage or putative sequence errors causing frame shift mutations were not included in the analysis. We validated the *in silico *mining method by aligning EST mined unigenes with four full length cDNA clones of grass IRI-like genes from the NCBI core nucleotide collection (barley; AK252915, AK249041/wheat; AY968588, AY968589).

### BAC identification and sequencing

Two perennial ryegrass BAC libraries were used to identify novel IRI-like genes [45]. Primers for the initial identification of novel IRI-like genes were designed from coding sequences of *LpAFP *(AJ277399) and a partial sequence of a *Festuca pratensis *IRI-like homolog (EU684537). The LpAFP primer pair had forward primer 5'GATGAACAGCCGAATACGATTTCT3' and reverse primer 5'GCTTCCAGATACAACGTGGTTGCT3', denaturing at 94°C for 4 minutes and then 35 cycles of 94°C 30 s, 60°C 45 s, 72°C 45 s, and 72°C 10 min. Primer pairs designed from the *F. pratensis *sequence were forward primer 5'TGTCATATCGGGGAACAACA3' and reverse primer 5'ACATGGTTTCGTCCGGATAC3' denaturing at 94°C for 4 minutes and then 40 cycles of 94°C 10 s, 60°C 45 s, and 72°C 1.30 min, and 70°C for 10 min. We also designed a third primer pair, referred to as LpIRIx primer pair, with forward primer 5'GAATGCCGTATCTGGGGACC3' and reverse primer 5'GTGGTTCCCGGATACGGTATT3', based on multiple sequence data acquired from sequencing of the above mentioned genes. This primer pair was used under the same conditions as the LpAFP primers. DNA maxi-preps of the BAC-clones were preformed using the NucloBond BAC 100 Kit (MACHEREY-NAGEL, Düren, Germany). For BAC-sequencing 500 ng BAC-DNA was combined with 20 μM of primer, 8 μl BigDye 3.1 ready mix and dH_2_0, to 20 μl total volume. Following 5 min of denaturising at 95°C, 50 cycles were performed with 30 s at 95°C, 10 s 50°C, and 4 min 60°C. Subsequently, the sequencing reactions were precipitated and sequenced on an ABI PRISM 3100 (Applied Biosystems, Foster City, CA, USA).

### Protein domain characterisation

Predicted peptide LRR domains were characterised using Pfam [46]. We verified the Pfam results by visual inspection of the sequences defining a LRR motif as LxxLxLxx, or variations of it where L is substituted with I, V, or A. To track the molecular evolution of the LRR-domain, *OsLRR-PSR *was used as a template for comparison to the predicted domains of IRI-like sequences. LRR motifs predicted by Pfam were considered significant if the Pfam E-value was lower than 0.05. IRI-like amino acid sequences were aligned with the LRR-domain of OsLRR-PSR and the LRR motifs in IRI-like sequences were named according to which of the LRR motif number in OsLRR-PSR they aligned to. IRI-domain characterisation was performed by visual scoring of the total number of repeat motifs (NxVxG/NxVxxG). IRI-domain repeat motifs were considered as "present", and counted, when they contained no more than one amino acid substitution compared to the consensus motifs. Signal peptide domains were predicted by TargetP [47].

### Estimation of substitution rates

To estimate divergence times between putatively paralogous and orthologous sequences we used the average dS obtained from three different methods, Nei & Gojobori [48], Kumar [49], and the Li-Wu-Luo [50] method, in MEGA (4.0) [51]. As a control for evolutionary rates of IRI-like genes we calculated the average dS values of ten randomly selected orthologs from wheat, perennial ryegrass, and rice. Maximum likelihood estimation of non-synonymous to synonymous substitution ratios (w) was performed using Codeml in the PAML software package (v 3.15) [52]. The 3 × 4 codon substitution model was chosen for Codeml w estimations. PAL2NAL [53] was used to make codon based nucleotide alignments for the use in MEGA and PAML. The absolute time of divergence between orthologs and paralogs was estimated using a rate of 6.5*10^-9 ^substitutions/synonymous site/year for grasses [23]. For estimation of mutation rates and absolute divergence times we used the relationship k = dS/2T, where k is the absolute rate of synonymous substitutions per year, dS is the synonymous substitution rate, and T is the absolute time since divergence. To identify putative alleles not grouped in the same contig due to methodological errors, a cut-off threshold of dS = 0.03 was used. This threshold was set on the basis of average inter-allelic dS for LRR-domains of 27 disease resistance like genes in *A. thaliana *[54], and inter-allelic dS_max _of *LpIRI1 *calculated from twelve European perennial ryegrass genotypes (dS_max _= 0.015, data not published).

### Molecular and phylogenetic analysis

All amino acid and nucleotide alignments were made by MAFFT [55] and manually edited in BioEdit [56], and the phylogenetic trees were constructed in Treefinder [57]. An AIC criteria test [58], implemented in the Modeltest option in Treefinder, was used to choose substitution model for the phylogenetic analysis. ML trees were bootstrapped with 1000 replicates. Synonymous distance based trees were inferred by UPGMA from a pairwise dS distance matrix in MEGA. Alignment figures were prepared by BoxShade http://www.ch.embnet.org/software/BOX_faq.html.

## Authors' contributions

SRS conceived the study, carried out molecular genetics work and molecular analysis, and drafted the manuscript. HR carried out molecular genetics work, helped with data analysis, and participated in manuscript drafting. TA carried out molecular work and helped draft the manuscript. OAR participated in drafting the manuscript.

## Supplementary Material

Additional file 1 EST accession numbers. Table giving all EST accession numbers included in the full length IRI-like *in silico *mined sequences. Click here for file

Additional file 2 Amino acid alignment of perennial ryegrass IRI-like sequences. Amino acid alignment of perennial ryegrass IRI-like sequences used for phylogenetic analysis. Click here for file

Additional file 3 Amino acid alignment of wheat IRI-like sequences. Amino acid alignment of wheat IRI-like sequences used for phylogenetic analysis. Click here for file

Additional file 4 Amino acid alignment of barley IRI-like sequences. Amino acid alignment of barley IRI-like sequences used for phylogenetic analysis. Click here for file
