# Supplementary material for: Tracking the evolution of a cold stress associated gene family in cold tolerant grasses
Source: BMC Evol Biol. 2008 Sep 5;8:245. doi: 10.1186/1471-2148-8-245 (PMC2542378; doi:10.1186/1471-2148-8-245)
Supplement: Additional file 1 — EST accession numbers. Table giving all EST accession numbers included in the full length IRI-like in silico mined sequences. [file 1471-2148-8-245-S1.pdf]

EST contig	EST contig accesion numbers												
TaC1	BE490074	CJ588184	CJ599857	CJ603376	CJ694370	CJ705367	CJ705367	CJ708689	CJ708697	CV762564	CV772016	CV773997	CV779975
	CV782708	DR740347	DR741812										
TaC2	BJ314975	CA701429	CJ597046	CJ598753	CJ600713	CJ600905	CJ601177	CJ601604	CJ602395	CJ603454	CJ603721	CJ603833	CJ604526
	CJ604676	CJ604939	CJ702804	CJ702804	CJ703666	CJ704377	CJ706336	CJ706607	CJ707005	CJ707732	CJ708765	CJ709227	CJ709329
	CJ709329	CJ709754	CJ709888	CJ710129	CK152036	CV759508	CV761182	CV769038	CV773203	CV778070	CV780144	CV780534	DR738876
TaC3	BE490254	BF200590	BF474043	BI479842	BJ224369	BQ166227	CJ589575	CJ603506	CJ695694	CJ707855	CJ709023	CJ709261	CJ710442
	CJ765575	CK197682	CV772213	CV773112	DY742123	DY742648							
TaC4	BE489991	BE518144	BQ161163	CJ597574	CJ599268	CJ603351	CJ603352	CJ703304	CJ704822	CJ708663	CJ708664	CJ905398	CJ905693
	CJ917110	CJ917628	CV765043	CV774448	CV777200	CV778490	DR739621	DR741024					
TaC5	CA701027	CJ588068	CJ592852	CJ597942	CJ601647	CJ604251	CJ694259	CJ698813	CJ703654	CJ707042	CJ709511	CK204475	CV758625
	CV774727	CV775065	DR739497										
TaC6	CA701727	CJ591530	CJ597029	CJ597169	CJ599965	CJ602185	CJ697568	CJ702789	CJ702799	CJ702918	CJ705466	CJ707531	
TaC7	AL821611	CJ600727	CJ601164	CJ603769	CJ606428	CJ706175	CJ706595	CJ708574	CJ708653	CJ709272	CJ711498	CV758718	CV765204
	CV766803	CV773908	OV766991										
TaC8	CA654225	CA692123	CJ587606	CJ588134	CJ596938	CJ598011	CJ600484	CJ693833	CJ694320	CJ702702	CJ703718	CJ705945	CJ913399
	CK196896	CK197231	CK205150	CV778647									
TaC9	CJ602439	CJ605514	CJ710676										
TaC10	CJ597869	CJ606637	CJ703587	CJ711694									
TaC11	CJ603876	CJ709369											
HvC1	AJ460319	AJ460320	AJ460321	AJ460322	AJ460323	AJ460324	AJ460325	AJ466155	BE411013	BF621173	BJ453528	BJ453816	BJ461352
	BM444259	BY847463	BY849668	BY868623	CA003455	CA003629	CA003758	CA004894	CA007651	CA009072			
HvC2	AV909087	AV909486	AV910970	AV911379	BJ447068	BJ447899	BJ448689	BJ448772	BJ449180	BJ450004	BJ450137	BJ450698	BJ451048
	BJ451602	BJ451821	BJ453251	BJ453323	BJ454200	BJ454271	BJ454431	BJ455071	BJ455644	BJ456413	BJ456641	BJ457114	BJ457425
	BJ457564	BJ458121	BJ458554	BJ459099	BJ459320	BJ459722	BJ460799	BJ460867	BJ461731	BJ461803	BJ461908	BJ462238	BY868126
	BY868350												
HvC3	BY836175	BY836245	BY836317	BY836782	BY837301	BY837351	BY837813	BY837818	BY837835	BY838037	BY838069	BY838097	BY840347
	BY841876	BY842473											
