# Supplementary material for: Tracking the evolution of a cold stress associated gene family in cold tolerant grasses
Source: BMC Evol Biol. 2008 Sep 5;8:245. doi: 10.1186/1471-2148-8-245 (PMC2542378; doi:10.1186/1471-2148-8-245)
Supplement: Additional file 2 — Amino acid alignment of perennial ryegrass IRI-like sequences. Amino acid alignment of perennial ryegrass IRI-like sequences used for phylogenetic analysis. [file 1471-2148-8-245-S2.pdf]

```
LpIRI1     1 MG---LLLLFLGFLLPAACAATSS-CHPDDLRALRGFAKNVGGGGVL-LRTAWSGTSCCV
LpIRI2     1 ------------------------------------------------------------
LpIRI3     1 MAKCLMLLLSFAFLLSAAGTATATPCHRDDLRALRGFAENLGGGGALSLRAAWSGASCCD
LpIRI4     1 MAKCWQLLLFLALLLPAASAAS---CHPDDLYALRDFAGNLRGGGVL-LRAALPGASCCG

LpIRI1    56 WEGVGCNGASGRITSLWLPRRGLAGTITGASLAGLAGLESLNLANNRLVGTIPSWIGELD
LpIRI2     1 ------------------------------------------------------------
LpIRI3    61 WEGVGCDGASGRVTALWLPRSGLT-------------------------GPIPSWICQLH
LpIRI4    57 WEGVGCDGASGCVKSFQIL-----------------------------------------

LpIRI1   116 HLLYLDLSHNSLVGELPNRLRIRLKGLTTTGHLLGMTFTNMPLDVKHNRRTLAIQPNTIS
LpIRI2     1 ----------------------------------------MPLHVKRSQGTLDEDHNTIT
LpIRI3    96 HLRYLDLSGNALVGEVPKNLQVQLKG-----------ITNMPLHVMRNRRSLDEQPNTIS
LpIRI4    76 -----------------------LKGLTAAGRSLGKAFTHMPLHVKPSQGTLDEDHNTIT

LpIRI1   176 GTNNLVLSGRNNVVSGNDNTVISGNNNTVSGSFNTVVTGSDNILTGSNHVVSGRSHIVTD
LpIRI2    21 GSHNTVRSGSNNVVSGNDNTVISGNNNVMSGSHNTVIFGGDNFVSGSYHVVSGNHHVVTD
LpIRI3   145 GSNNTVRSGSKNVLAGNDNTVISGDNNSVSGSNNTVVSGNDNTVTGSNHVVSGTNHIVTD
LpIRI4   113 GINNTVRSGSNNVVSGNDNTVISGNNNVVSGSHNTVVFGGDNFISGSYHVVSGNHHVVTD

LpIRI1   236 NNNSVSGDDNNVSGSFHKVSGSHNTVSGSNNTVSGRN---------------------HV
LpIRI2    81 NKNAVSGDHNTVSGTQNTVSGNHQIVSGSHGTVSGNHNTVSGRNNSVYGNKNIVSGSNHV
LpIRI3   205 NNNNVSGNDNNVSGSFHTVSGGHNTVSGSNNTVSGSN---------------------HV
LpIRI4   173 NKNAVSGDHNTVSGSQNTVSGNHQIVSGSHSTVSGNHNTVSGRNNSVYGNNNIVSGSNHV

LpIRI1   275 VSGSNKVVTGG
LpIRI2   141 VYGNNKVVTGG
LpIRI3   244 VSGSNKVVTDA
LpIRI4   233 VYGNNKVVTGG
```
