# Supplementary material for: Tracking the evolution of a cold stress associated gene family in cold tolerant grasses
Source: BMC Evol Biol. 2008 Sep 5;8:245. doi: 10.1186/1471-2148-8-245 (PMC2542378; doi:10.1186/1471-2148-8-245)
Supplement: Additional file 3 — Amino acid alignment of wheat IRI-like sequences. Amino acid alignment of wheat IRI-like sequences used for phylogenetic analysis. [file 1471-2148-8-245-S3.pdf]

```
TaC1      1 MAKCGLLFLFLAFLLPAARATSCHPDDLRALRGFAGNLSGGAVLLRAAWSGASCCVWEGVSCDGTSGRVTALRLPGHGLV
TaC5      1 MAKCGLLILFLGFLLAAARATSCHLDDLRALRGFAGNLSGGAVLLRAAWSGASCCGWEGVSCDGTSGRVTALRLPGHGLV
TaC3      1 MAKCGLLLLFFAFLLPAARATSCHPDDLRALRGFAGNLSGGAALLRAAWSGASCCVWEGVNCDGTSGRVTALRLPGHGLV
TaC4      1 MAKCWLMLLFLVFLLPATSATSCHADDLRALRDFARNLTGGGVILRAAWSGTWCCRWEGVGCDGRSGRVTTLRLPGRGLA
TaC2      1 MAKCWLLLQFLAFLLPATSATSCHTDDLRALQGFAGNLSGGGVLLRAVWTGVSCCGWEGVSCDGTSGRVTGLRLPGRGLA
TaC6      1 MAKCWLLLQFLAFLLPAASATSCHADDLRALQGFAGNLSGGGVLLRAVWTGVSCCGWEGVSCDGTSGRVTGLRLPGRGLA
TaC11     1 MAKCWLLLQFLAFLLPATSATSCHTDDLRALQGFAGNLSGGGVLLRAVWTGVSCCGWEGVSCDGTSGRVTGLRLPGCGLA
TaC7      1 MAKCWLLLHLLAFLLPAASATSCHTDDLHALQGFAGNLGGGGVLLRAVWSGASCCGWEGVSCDGTSGRVTALRLPGHGLA
TaC8      1 MAKCWLLLHLLAFLLPAASATSCHTDDLHALQGFAGNLGGGGVLLRAVWSGASCCGWEGVSCDGTSGRVTALRLPGHGLV
TaC9      1 MAKCWLLLHFLAFLLPPASATTCHADDLRALRGFAGNLSGGGVLLRAMWSGASCCGWEGVGCDGQSGRVMALLLPGRGLA
TaC10     1 MAKCWLLLHFLAFVLPAASATTCHSDDLRALRGFAGNLSGGGVLLRTTWSGALCCGWEGVGCDGQSGRVTALLLPGRGLA
TaIRI2    1 MAKSWLLLFCLAFLLPAASAT-CHPDDLRALRGLAGTLGAVARHLHSVWSGASCCDWEGVGCHGASGRVTALRLHGHGLA

TaC1     81 GPIPGASLAGLARLEEL---------------------------------------------------------------
TaC5     81 GPIPGASLAGLARLEEL---------------------------------------------------------------
TaC3     81 GLIPGASLAGLARLEEL---------------------------------------------------------------
TaC4     81 GPIAGASLAGLARLEEL---------------------------------------------------------------
TaC2     81 GPIPRASLAGLAQLEEL---------------------------------------------------------------
TaC6     81 GPIPRASLAGLVQLEEL---------------------------------------------------------------
TaC11    81 GPIPRASLAGLAQLEEL---------------------------------------------------------------
TaC7     81 GPISGASLAGLTQLVEL---------------------------------------------------------------
TaC8     81 GPIPGASLAGLTQLVEL---------------------------------------------------------------
TaC9     81 GPIAGASLAGLVQLEEL---------------------------------------------------------------
TaC10    81 GPIPGASLAGLVQLEEL---------------------------------------------------------------
TaIRI2   80 GPIPGASLAGLIRLEELFLGSNSFAGTLPDELFSLAGLQRLSLASNNLAGQLSSRLSDLINLTLLDLSINHFSGHLPDNV

TaC1     98 --------------------------------------------------------------------------------
TaC5     98 --------------------------------------------------------------------------------
TaC3     98 --------------------------------------------------------------------------------
TaC4     98 --------------------------------------------------------------------------------
TaC2     98 --------------------------------------------------------------------------------
TaC6     98 --------------------------------------------------------------------------------
TaC11    98 --------------------------------------------------------------------------------
TaC7     98 --------------------------------------------------------------------------------
TaC8     98 --------------------------------------------------------------------------------
TaC9     98 --------------------------------------------------------------------------------
TaC10    98 --------------------------------------------------------------------------------
TaIRI2  160 FRDLMSLEHLVAHSNGFSGSLPPSLSSLSSLRELNLRNNSLSGPISHVNFSGMPLLASVDLSTNYLNGLLPVSLADCGEL

TaC1     98 ---NLANNKLVGTIPSWIGELDHLCYLDLSDNSLVGEVPKSLIRLKGLVI-VGRSLGMVFTNMPLYVKRNRRTL-DE--Q
TaC5     98 ---NLANNKLVGTIPSWIGELDHLCYLDLSDNSLVGEVPESLIQLKGLVI-AGRSLGTAFTNMPLYVKSNRRTL-DE--Q
TaC3     98 ---NLANNKLVGTIPSWIGELDHLCYLDLSDNSLVGEVPKSLIRLKGLVI-AGHSLGMVFTNMPLYVKRNRRTL-DE--Q
TaC4     98 ---NLANNRLIGTFPSWIGELEHLRYLDLSDNSLIGEVPKSLIRFKDITI-AGRSLGKAFTNMPLYVKSNRRTL-QQQPQ
TaC2     98 ---NLANNKLIGTIPSWIGELDHLCYLDLSDNLLVGEVSKSLIQLKGFAT-TGRSLGMAFTNMPLYVKRSRRTL-QQQ-Q
TaC6     98 ---NLASNKLIGTIPLWIGELDHLCYLDLSDNLLVGDVPKSLIQLKGFAT-IGCSLGMAFTNMPLYGKHSRRTL-QQQ-Q
TaC11    98 ---NLANNKLIGTIPSWIA-------------------------------------------------------------
TaC7     98 ---NLANNKLIGTISSWIGELDHLRYLDLSNNLLVDEVPKRLIQLKGLAS-TGRSLG------------NRRTL-QQQ-Q
TaC8     98 ---NLANNKLIGTIPSWIGELDHLCYLDLSNNSLVGEVPKTLIQLKGLVS-TGRSLG------------NRRTLQQQQ-Q
TaC9     98 ---NLSNNKLIGTVPSWIGELDHLCYLDLSDNLLVGKVPKNLINLKGLAT-TGRLMGMAFTSMPLHVMRNRRIL-QQQ-R
TaC10    98 ---NLSNNQLIGTIPSWIGELDHLRYLDLSGNSLVGEVPKSLINLQGLAT-IGRLLGMAFTSMPLHMMRNRRTL-QQQ-R
TaIRI2  240 RSLSLANNRLVGTIPSWIGELDHLHYLDLSNNSMIGKVPKSSTRLKGLATVVGHSPGMAFTNMPLYEKRNRRIL-GQ--Q

TaC1    171 PNTISGSNNTVRSGSTNVVSGNDNTVISGNNNNVAGSNNTVVTGNNNTVTGSNHIVSGDKHIVTDNNNAVSGNDNNVSGS
TaC5    171 PNTITGSNNTVRSGSTNVVSGNDNTVISGNNNNVAGSNNTVVTGNDNTITGSNHVVSGNKHIITDNNNAVSGNDNNVSGS
TaC3    171 PNTISGSNNTVRSGSTNVVSGNDNTVISGNNNNVAGSNNTVITGNDNTVTGSNHVVSGDKHIVTDNNNAVSG--------
TaC4    173 PNTISGTNNTVRSGSTNVVSGNDNTVISGNNNNVSGSNNTIVTGSDNTVVGSNHVVSGNKHVVTDNNNAVSGNDNNVSGS
TaC2    172 PNIISGTNNKVRSGRTNVVSGNDNTVVSGNDNTVAGSNNTITTGSGNTVTGSNHVVSGTKHIVTDNNNVVSGIDNNVSGS
TaC6    172 PNIISGTNNKVRSGRTNVVSGNDNTVVSGNDNTVAGSNNTITTGSGNTVTGSNHVVSGSKHIVTDNNNVVSGIDNNVSGS
TaC11       --------------------------------------------------------------------------------
TaC7    160 PNIISGTNNKVRSGRTNVVSGNDNTVISGNNNTVAGSNNTITTGSDNTVTGSNHVVSGSKHIVTDNNNVVSGIDNNVSGS
TaC8    161 PNIISGTNNKVRSGRTNVVSGNDNTVISGNNNTVAGSNNTITTGSDNTVTGSNHVVSGSKHIVTDNNNVVSGIDNNVSGS
TaC9    172 PNIISGTNNKVRSGRTNVLSGNDNTVIFGNSNTVAGSNNTITTGSDNTVTGSNHIVSGSKHIVTDNNDVVSGIDNNVSGS
TaC10   172 PNIISGTNNKVRSGRTNVLSGND---------------------------------------------------------
TaIRI2  317 PNVISGSRNTIRSGTNNVLSGNDNTVISGN---------------DNVVTGSNNVVSGSGNVVTERNHVVSGSDN-----

TaC1    251 FHTVSGSHNTVSGTNNTVSGSNHVVSGSNKVV-GDQ
TaC5    251 FHTVSGSRNTVSGTNNTVSGSNHVVSGSNKVV-GDE
TaC3        ------------------------------------
TaC4    253 FHTVSGSRNTVSGSNNTVSGSNHVVSGSNKVVTGDE
TaC2    252 FHTVSGTLNTVSGSNNTVSGSNHVVSGSNKVVTGG-
TaC6    252 FHTVSGSHNTVSGSNNTVSGSNHVVSGSNKVVTGG-
TaC11       ------------------------------------
TaC7    240 FHTVSGSHNTVSGSNNTVSGSNHVVSGSNKVVTGG-
TaC8    241 FHTVSGSHNTVSGSNNTVSGSNHVVSGSNKVVTGG-
TaC9    252 FHTVSGSHNTVSGSNNTVSGSNHVVSGSNKVVTGG-
TaC10       ------------------------------------
TaIRI2  377 --VVSGRNNVVTGSNNVVSGRNHVVSGNNKVVTGG-
```
