# Supplementary material for: Tracking the evolution of a cold stress associated gene family in cold tolerant grasses
Source: BMC Evol Biol. 2008 Sep 5;8:245. doi: 10.1186/1471-2148-8-245 (PMC2542378; doi:10.1186/1471-2148-8-245)
Supplement: Additional file 4 — Amino acid alignment of barley IRI-like sequences. Amino acid alignment of barley IRI-like sequences used for phylogenetic analysis. [file 1471-2148-8-245-S4.pdf]

```
HvC2        1 MAKCGLLLLF-LAFLLPAAGATSCHVDDLRALRGFAGNLSGGAVL-LRAA
HvC1        1 MAKYWMLLHF-LAVLLPAARATSCHVDDLRALRDFAGNLSGGGAL-LRAV
HvC3        1 MAKCWLLLLC-LAFLLPAACAT-CHPDDLRALRGLARNLRGGGAVHLRTV
AK249041    1 MAKCCLLLAFLLAFLLPVAYAT-FHPDDLHALRGSAGKI-----------

HvC2       49 WSGASCCGWEGVGCDGPSGRVTSLRLPGHDLGGAIPGASLAGLAWLEELN
HvC1       49 WSGTSCCGWEGVGCDGASGRVTTLRLPGRGLVGRIPGASLAGLAWLQELN
HvC3       49 WSGASCCDWEGVGCHGANGRVTVLRLPGHGLAGSIPGASLAGLARLEELS
AK249041   39 --------------------------------------------------

HvC2       99 LANNRLVGTIPSWIGELDHLYYLDLSDNSLVGEVPKSLIRLKGFAIAGRS
HvC1       99 LASNRLVGTIPSWIGELDHLCDLDLSGNSLDGEVPKSLIRLKGHAAAGRS
HvC3       99 LSSNSFAGTLPDALFGLVGLRKL---------------------------
AK249041   39 --------------------------------------------------

HvC2      149 SGMIFTNMPLYVEPNRRML-DEQPNTISGSNNTVRSGSTNVVSGNDNTVI
HvC1      149 SGMTFTNMPLYVKRNRRTLQQQQPNIISGTNNKVRSGRNNVVSGNDNAVI
HvC3          --------------------------------------------------
AK249041   39 --------------NRRTLQQQQPNTITGTNNSVRSGSGNIISGSGNTVV

HvC2      198 SGNNNNVAGSNNTIVTGNDNTVTGSNHVVSGDKHIVTDNNNAVSGNDNNV
HvC1      199 SGNNNTVAGSNNTITTGSDNTVTGSNHVVSGSKHIVTDNNNVVSGIDNNV
HvC3          --------------------------------------------------
AK249041   75 SGDNNNISGSNNTVTSGSNNVIVNTNHVVTGSNN----------------

HvC2      248 SGSFHTVSGSHNTVSGTNNTVSGNNHVVSGSNKVVGDA
HvC1      249 SGSFHTVSGSLNTVSGSNNTVSGSNHVVSGSNKVVTGG
HvC3          --------------------------------------
AK249041  109 -----TVSGNNNRVTGNNNIVSGSNQVVSGDNKVVTG-
```
